# Living with frailty and haemodialysis: a qualitative study

**DOI:** 10.1186/s12882-022-02857-w

**Published:** 2022-07-22

**Authors:** Hannah M. L. Young, Nicki Ruddock, Mary Harrison, Samantha Goodliffe, Courtney J. Lightfoot, Juliette Mayes, Andrew C. Nixon, Sharlene A. Greenwood, Simon Conroy, Sally J. Singh, James O. Burton, Alice C. Smith, Helen Eborall

**Affiliations:** 1 Leicester Diabetes Centre, University Hospitals of Leicester NHS Trust, Leicester, England; 2 Department of Research and Innovation, University Hospitals of Leicester NHS Trust, Leicester, England; 3 Department of Respiratory Sciences, University of Leicester, Leicester, England; 4 Leicester Diabetes Centre, Leicester General Hospital, Gwendolen Road, Leicester, England; 5 John Walls Renal Unit, University Hospitals of Leicester NHS Trust, Leicester, England; 6 School of Health, Wellbeing and Social Care, The Open University, Milton Keynes, England; 7 Department of Health Sciences, University of Leicester, Leicester, England; 8 Department of Physiotherapy and Renal Medicine, King’s College Hospital and Department of Renal Medicine, King’s College London, London, England; 9 Department of Renal Medicine, Lancashire Teaching Hospitals NHS Foundation Trust, Preston, UK; 10 Central and North West London NHS Foundation Trust - MRC Unit for Lifelong Health and Ageing, University College London, London, England; 11 Centre for Exercise & Rehabilitation Science, NIHR Leicester Biomedical Research Centre, Leicester, England; 12 Department of Respiratory Medicine, University Hospitals of Leicester, Leicester, England; 13 Department of Cardiovascular Sciences, University of Leicester, Leicester, England; 14 National Centre for Sport and Exercise Medicine, Loughborough University, Loughborough, England; 15 Usher Institute, University of Edinburgh, Edinburgh, Scotland

**Keywords:** Haemodialysis, Frailty, Dialysis, Multi-morbidity, Qualitative, Interviews, Patient experience

## Abstract

**Background:**

Frailty is highly prevalent in people receiving haemodialysis (HD) and is associated with poor outcomes. Understanding the lived experiences of this group is essential to inform holistic care delivery.

**Methods:**

Semi-structured interviews with *N* = 25 prevalent adults receiving HD from 3 HD units in the UK. Eligibility criteria included a Clinical Frailty Scale (CFS) score of 4–7 and a history of at least one fall in the last 6 months. Sampling began guided by maximum variation sampling to ensure diversity in frailty status; subsequently theoretical sampling enabled exploration of preliminary themes. Analysis was informed by constructivist grounded theory; later we drew upon the socioecological model.

**Results:**

Participants had a mean age of 69 ± 10 years, 13 were female, and 13 were White British. 14 participants were vulnerable or mildly frail (CFS 4–5), and 11 moderately or severely frail (CFS 6–7). Participants characterised frailty as weight loss, weakness, exhaustion, pain and sleep disturbance arising from multiple long-term conditions. Participants’ accounts revealed: the consequences of frailty (variable function and psychological ill-health at the individual level; increasing reliance upon family at the interpersonal level; burdensome health and social care interactions at the organisational level; reduced participation at the community level; challenges with financial support at the societal level); coping strategies (avoidance, vigilance, and resignation); and unmet needs (overprotection from family and healthcare professionals, transactional health and social care exchanges).

**Conclusions:**

The implementation of a holistic needs assessment, person-centred health and social care systems, greater family support and enhancing opportunities for community participation may all improve outcomes and experience. An approach which encompasses all these strategies, together with wider public health interventions, may have a greater sustained impact.

**Trial registration:**

ISRCTN12840463.

**Supplementary Information:**

The online version contains supplementary material available at 10.1186/s12882-022-02857-w.

## Background

In the haemodialysis (HD) population, the prevalence of frailty, “a multidimensional syndrome of decreased physiological reserve leading to increased vulnerability to minor health stressors” [1], is 34% [2], compared with 7.7% in older adults in the general population [3]. People receiving HD are particularly susceptible to developing frailty irrespective of chronological age; this is associated with an increased risk of mortality [4]; hospitalisation [5]; falls [6] and loss of independence and quality of life [7, 8].

HD treatment was originally intended for a more robust demographic with lower levels of multimorbidity [9]. Debate about appropriate care for a frailer population has begun to emerge [10], yet there are relatively few studies of interventions which might improve outcomes, care, and experience for this vulnerable group [11–14]. Studies examining the impact of frailty on people receiving HD have primarily used observational designs. To identify and/or develop interventions and strategies to improve experiences and outcomes for this group, a better understanding of their holistic needs is essential.

While several qualitative studies have focused upon the experience of older adults in relation to specific aspects of care or HD treatment [17–21], there has been little examination of the wider experiences of people who are living with frailty, irrespective of their age. This paper reports on a qualitative exploration of the lived experiences of people receiving HD who are frail, which aimed to identify what factors should inform the care of this group.

## Methods

### Background context

This paper reports on a qualitative study, which was part of a larger programme of research (Table 1). In this paper, we concentrate specifically on participants’ experiences of living with frailty, using data from in-depth interviews.

**Table 1:** Background context surrounding this study

**Background: FLEX-HD feasibility trial and qualitative studies**
The current qualitative study was embedded in a larger programme of research [22] which aimed to: - determine whether intradialytic cycling (IDC, cycle ergometry delivered during HD treatment using a bespoke static exercise bike) was feasible for people living with frailty and receiving HD - inform a tailored exercise intervention for this population • Inclusion criteria for the qualitative studies: - receiving HD for 3 months - aged 18 years or older - classified as vulnerable to severely frail according to the Clinical Frailty Scale (CFS score 4–7) - able provide informed consent and speak English - with a history of at least one fall in the last 6 months Who had both declined to, and had participated in, IDC. • The qualitative studies involved three stages of data collection: - semi-structured interviews exploring participants experience of living with HD, frailty and falls - self-completed diaries gathering contemporaneous information about their experiences of falls over a period of up to three months - follow-up interviews exploring diary entries, experiences of participating in a trial (or reasons for declining), participants perceptions of IDC and their needs relating to exercise.

Reporting follows the consolidated criteria for reporting qualitative research (COREQ) [23]. Ethical approval was granted by the NHS Research Ethics Committee Southwest (Bristol; REC ref: 17/SW/0048) and the study prospectively registered (study registration number ISRCTN12840463).

### Participant selection

Participants were initially selected using maximum variation sampling to maximise diversity in frailty status. Analysis occurred in parallel with data collection; preliminary analyses later informed theoretical sampling to further recruit participants who had characteristics which could extend the existing themes [24].

Inclusion criteria were participants: (a) receiving HD for 3 months, (b) aged 18 years or older, (c) classified as vulnerable to severely frail according to the Clinical Frailty Scale (CFS score 4–7), (d) able provide informed consent and speak English and, (e), with a history of at least one fall in the last 6 months [25].

Exclusion criteria included being: (a) unable or unwilling to give informed consent (b) unable to understand, read or speak English and, given the broader study outlined above, being (c) unable to participate in an exercise due to perceived physical or psychological barriers or (d) unable to undertake exercise or exercise testing according to the American College of Sports Medicine guidelines [26].

Participants were recruited from three haemodialysis centres (1 hospital-based HD unit and 2 satellite units) within the East Midlands in the UK between May 2017 and January 2019. Potential participants were approached during HD. All participants provided written informed consent. Recruitment ceased at the point at which new insight was no longer generated.

### Data collection

Semi-structured interviews provided a flexible framework from which to explore individuals’ experiences. A topic guide was developed by HMLY, HE and the patient and public involvement (PPI) group, following a review of the literature. Discussion with the PPI group highlighted that people who met the criteria for frailty did not identify themselves as such, and the term elicited strongly negative reactions. Consequently, the term ‘frailty’ was avoided in interviews. The topic guide (Supplementary Material 1) was piloted and refined during the first three interviews.

HMLY, an experienced qualitative researcher and physiotherapist, conducted the interviews during HD treatment or in the participant’s home, dependent on preference. Interviews on the HD unit were conducted behind screens or curtains or in a side-room, where possible, to allow more privacy and to encourage open discussion. HMLY was known to some of the participants, although not directly involved in their clinical care. Interviews lasted 30–120 (mean 62) minutes. Fieldnotes were made after each interview. All interviews were audio-recorded, transcribed verbatim and anonymised. We present the findings from our conceptual analysis of participants’ accounts, but seek to retain their presence while doing so [24].

### Data analysis

Analysis was informed by the principles of constructivist grounded theory [24]. Specifically, transcripts of the initial ten interviews were read and re-read by HMLY. Line by line coding, was undertaken to identify preliminary themes which formed the basis of a coding framework (developed with MH and NR). As further data were collected, constant comparison with that previously collected helped to adapt, expand, or merge codes and/or themes [24]. Subsequent coding focused on themes most significant to the research aims [24]. A final phase of analysis ascribed an order to the focused themes; at this point we drew upon the socioecological model to understand how experiences were shaped by individual, interpersonal, organisational, community and societal influences [27, 28]. Throughout all stages, memos were used to provide a record of analytic decision making [24]. The findings were reviewed by the PPI group to enhance credibility and generate additional insight. NVivo software (QSR International, version 11) was used to manage the data.

## Results

Twenty six of 37 people approached were recruited. One participant died prior to data collection, leading to a final sample of 25. Ten participants were recruited from satellite unit 1, 9 from the hospital-based unit and 6 from satellite unit 2. Participants’ mean age was 69 ± 10 years, 13 were female and 13 were White British and 10 Asian or Asian British (see Table 2 for full sample characteristics). Interviews predominantly took place at the HD unit (*n* = 23).

**Table 2:** Participant demographics

		*N* = 25
**Age** (years)		69 ± 10
Sex, n (%)	Female	13 (52%)
	Male	12 (48%)
Ethnicity, n (%)	White British	13 (52%)
	Asian or Asian British	10 (40%)
	Caribbean	1 (4%)
	Not stated	1 (4%)
Diagnosis, n (%)	Diabetic nephropathy	11 (44%)
	Aetiology uncertain	6 (24%)
	Chronic pyelonephritis	3 (12%)
	Atypical hemolytic uremic syndrome	1 (4%)
	Focal segmental glomerulosclerosis with nephrotic syndrome	1 (4%)
	Henoch-Sconlein Purpura	1 (4%)
	Minimal change nephropathy	1 (4%)
	Polycystic kidney disease	1 (4%)
Charlson Co-morbidity Index		6 ± 2
Time on haemodialysis (months)		43 (IQR 16–85)
Number of medications		13 (IQR 10–16)
Clinical Frailty Scale (CFS) score, n (%)	CFS 4, Vulnerable	9 (36%)
	CFS 5, Mildly frail	5 (20%)
	CFS 6, Moderately frail	8 (32%)
	CFS 7, Severely frail	3 (12%)
Number of falls in last six months		3 (IQR 2–4)
Previous transplant, n (%)	No	21 (84%)
	Yes	4 (16%)
Active on transplant list, n (%)	No	22 (88%)
	Yes	3 (12%)
Employment status, n (%)	Retired	21 (84%)
	Unemployed	3 (12%)
	Part-time employed	1 (4%)
Marital status, n (%)	Married	15 (60%)
	Single	5 (20%)
	Widowed	5 (20%)
Social circumstances, n (%)	Lives with spouse or partner	11 (44%)
	Lives alone	9 (36%)
	Lives with extended family	5 (20%)^a^

Data are mean ± standard deviation or median (IQR) unless otherwise indicated

^a^ Demographic characteristics were extracted from participants medical records, Clinical Frailty Scale scoring was undertaken by the participants consultant nephrologist and information on falls and social circumstances were gathered from the participants

**Fig. 1:**
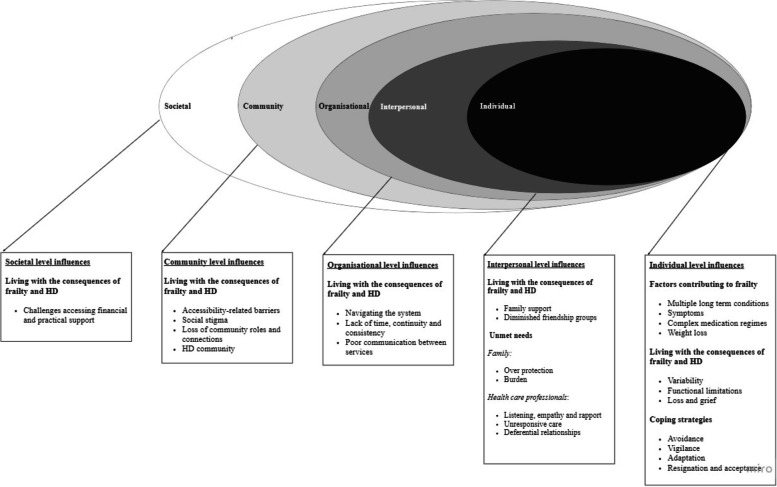
A socioecological model of living with frailty and receiving HD, with examples of key themes at each level

To present the findings, we begin with the background context of participants’ perceptions of the factors that contribute to frailty (illustrative quotes are presented in Table 3). Then, drawing upon the socioecological model, we describe participants’ experiences of the consequences of frailty – at different levels (Table 4). Thirdly, we present participants’ accounts of how they cope with their frailty, and its consequences, highlighting unmet needs (Table 5). Figure 1 outlines how each of the themes map to each level within the socioecological model.

**Table 3:** Quotations to illustrate factors influencing frailty

**Multiple long term conditions** * • ‘Is there no part of my body that is functioning properly? Eye, heart, back, kidneys, even my foot has an ulcer on it!’* (Participant 8, female, moderately frail, age 70s).
**Exhaustion** *•* ‘I can't walk now. I am so tired so I can't walk or step. I just want to sit, you know.’ (Participant 19, male, mildly frail, age 70s).
**Sleep** * • ‘I don't particularly sleep well at night, and I do sleep in the daytime… I go to bed early say about 9 o’clock and then I probably wake up at 12 o’clock having had about 3 hours. And then I am tossing and turning for the rest of the night. I wake up in the morning feeling awful, awful.’* (Participant 15, female, moderately frail, age 80s).
**Weakness** * • ‘I can't go out; I just can't go out at all. I have got a walker I can use to go out at weekends but there is no point me using it because the problem is I have got a weak back, weak neck. I just can't stand up long enough to do a cup of tea or walk anywhere. I am not even strong enough to push a walker I just feel I am going to collapse in a heap.’* (Participant 22, female, vulnerable, age 50s).
**Pain management** * • ‘I wake up, I take five tablets and my insulin… I keep the antibiotics with me here because they are supposed to be taken either with food or just after so you can't take them at any old time. But the other tablets there is one lot I take in the morning and one lot I take in the evening. The…thyroid people have said 50 g one day, 75 the next. I have got the Clonazepam; I take that every day and Gabapentin…three times a week. And now they have changed my PRN ones…I have got to look every time’* (Participant 8, female, moderately frail, age 70s). * • ‘The highest painkiller I am allowed is paracetamol. My shoulder was in pain they said alright we will give you ibuprofen gel and it’s like putting face cream on really!’* (Participant 5, male, moderately frail, age 60s). * • ‘I find erm a bath helps, so warm water, we have an air bath, a jacuzzi. So that helps, being weightless in water, for a while anyway’* (Participant 13, male, vulnerable, age 50s).
**Weight loss** * • ‘When I’ve made [some food] it takes me an hour and I’m in absolute bloody agony at the end of it. Then when you taste it the first two mouthfuls are alright but then it’s just really bland and tasteless. My taste buds seem to have gone and you just think I’ve spent all that time and it just wasn’t worth it.’* (Participant 16, male, vulnerable, age 60s).

**Table 4:** Quotations to illustrate living with the consequences of frailty and haemodialysis

**Individual level**
** Variability** • *‘It definitely is day to day thing; some days are better than others.’ *(Participant 3, female, vulnerable, age 40s). ** Functional limitations** • *‘My bedroom is upstairs, so I only come out from my bedroom twice a day when I’m not on dialysis for two minutes in the morning and the evening. And then I go upstairs and then come out next day. I’m having a difficulty for the stairs.’* (Participant 11, male, severely frail, age 60s). • *‘I can’t raise my right hand above shoulder height. I can’t reach out and pick the hand-wash up, the strain on my shoulder, I just start trembling. The left one’s alright, if I need anything out the cupboard, I have to use the left one. Sometimes when I pick my cup of tea up, I have to support my right wrist with my left arm. If I reach for anything it’s always with the left.’* (Participant 16, male, vulnerable, age 60s). ** Loss and grief** • *‘I just want my normal life back. I don’t know what to expect with everything. It’s too much.’* (Participant 18, female, vulnerable, age 60s).
**Interpersonal level**
** Family support** • *“If I want to go anywhere I can manage to go in the car with either daughters…they always take me to the doctors, take me to the hospital, take me shopping. No matter where I want to go, there’d be one of them.*’ (Participant 14, female, moderately frail, age 80s). • *‘My husband is a lot stronger than me; he really is… and when I am not feeling too good he is there. Whenever I am ill like this I want him there.’* (Participant 4, female, severely frail, age 60s). ** Diminished friendship groups** • *‘I was just too social, I had plenty of friends….I am at home now, I don't go out at all. If I have to go out, only for the GP appointment. Otherwise, I prefer to stay at home.’* (Participant 11, male, severely frail, age 60s).
**Organisational level**
** Navigating the system** • *‘I have got a page full of…appointments. There is always something, I have got to go to the hospital on 27*^*th*^*, 28*^*th*^* and 29*^*th.*^ *My three appointments are for different things, and the same consultant won’t see you for different things because one is a diabetic specialist, kidney specialist and one is a something else specialist, so they don't see you for all three anyway.’* (Participant 5, male, moderately frail, age 60s). ** Lack of time, continuity and consistency** • *‘[Therapists] did come but they come one day, and they are there for half an hour and then they are ‘oh well we will see you in three weeks’ time.’ To be quite honest it’s no use nor ornament.’* (Participant 12, male, mildly frail, age 70s). • *‘It ends up being locums, there could be a doctor there, but he is never there, but they have plenty of locums. The trouble with locum doctors is sometimes you walk in, and they haven't read anything about you..’* (Participant 5, male, moderately frail, age 60s). ** Poor communication between services** • *‘[Orthopaedics] were reluctant to operate because they didn’t think my heart could take the strain. So, they set the operations up but then [the anaesthetist] would back out or cancel and that went on for a year and a half. The anaesthetist has the option of whether they chose to deal with that patient or not and they considered me really high risk. So, I would have meetings with them and afterwards it would be 'not yet' and 'in a while' and just keeping me in so much pain. I was on pain killers and a bottle of wine a night and then anything that would numb the pain.’* (Participant 16, male, vulnerable, age 60s).
**Community level**
** Accessibility-related barriers** • *‘We went to a cafe once, my friend [took me in a] wheelchair. We got to this cafe and there was a small step down, it wasn't big, she went in and said have you got a ramp and [the café owner] said yes, they were hoping to fit one but they hadn't got it, it was more like a half step than an actual ramp, but they assured us they were going to get a proper ramp.’*(Participant 8, female, moderately frail, age 70s). **Social stigma** • *‘I don’t feel like going out, because if you are not well, you can’t get dressed up nicely. I used to years ago I would dress up very nicely, saris and all that.’* (Participant 18, female, vulnerable, age 60s). • *‘The Zimmer frame I use that in the house. I use it when it’s around, [my wife] don't like using it, she never uses it when she is out, she is like me, she doesn't like people to think she is ill.’* (Participant 26, male, severely frail, age 80s). ** Loss of community roles and connections** • *‘This Indian dance, this folk dance with the sticks and all. My neighbours they used to call me, and I joined in. [I stopped] because we moved… it wasn’t the same. Leicester they have only in the club, they don’t have it at home like that… now I can’t, I’m older you see now. I can’t manage it; I get very tired’*. (Participant 7, female, mildly frail, age 80s). ** HD community** • ‘*The nurses here are ever so nice and if I think about it really the only contact I get is when I come in here [HD unit] and yet I hate what they do to me.’* (Participant 26, male, severely frail, age 80s).
**Societal level**
** Challenges accessing financial and practical support** • *‘All I wanted was a disabled badge so I could park the car and not have to walk for miles. But the council wouldn't give it to me because I wasn't on disability allowance. The doctors gave me a really good…letter to [explain that I am on] long term dialysis, but they still rejected it. So, I even thought if being on disability allowance is going to give me a badge then I will go on it. So, I went to the office got the forms and everything but … so much mail came through I said to my husband “forget it I don't want this”’* (Participant 10, female, vulnerable age 50s). • *‘The people that are making these [disability] assessments are…not qualified to make significant medical judgement. They do it from a set of questions that they’ve been given, and they follow it religiously…so when you say yes I can, tick. They don’t want to know the consequences of doing that action and the fact that you may be in pain, or you have fatigue or, you know, that you have to then rest for three or four hours or even sleep. They don’t consider those kind of problems, it’s yes or no’*(Participant 13, male, vulnerable, age 50s). • *‘I lost a lot of money from social security because I didn’t know that you’re supposed to be getting a single person’s disability money. Nobody’s bothered to look at what you’re supposed to be entitled to or how much you should be getting.’*(Participant 6, male, vulnerable age 50s).

**Table 5:** Quotations to illustrate coping strategies and unmet needs

**Individual level—coping strategies**
** Avoidance** • *I’ve stopped going to [the supermarket] because I struggled to get round. I have to push the trolley six feet in front of me and then catch it up. I used to be able to walk with one crutch and one hand on the trolley and get round quite easily. I can’t do that now, so you have to push the trolley and then catch it up and then put your stuff in and then push the trolley and catch it up. And you hear people saying oh look at that poor bloke over there, look at that poor old fella. Old ladies coming up and saying can I help you and things, and I should be saying that to them.’* (Participant 16, male, vulnerable, age 60s). ** Vigilance** • *‘Since the previous [fall] I try and have my mobile on me, and I decided to get one of these the alarm things.’* (Participant 12, male, mildly frail, age 70s). • *‘You think of what you are doing because if you are concentrating on what you are doing you are not going to do something like slip.’* (Participant 25, male, moderately frail, age 70s). ** Adaptation** • *‘I find different ways to do things… it might not be conventional, but I find ways.’* (Participant 23, female, moderately frail, age 60s). • *‘We’ve got lifts to get us in and out of the bath, lifts on the bed, grab rails everywhere, raised toilet seats with arms, a reclining chair. All sorts of things. Cars that have got adaptations with hoists to lift scooters.’* (Participant 13, male, vulnerable, age 50s). ** Resignation and acceptance** • *‘You have to get used to it when you are in this position you know. I don't like it, but you have got no choice.’* (Participant 15, female, moderately frail, age 80s). • *‘I have given up; I have given up hope.’* (Participant 2, male, moderately frail, age 70s).
**Interpersonal level—others responses to frailty**
** Over protection** • *‘My daughter warned me not to go upstairs. I went up once …I had got a rail each side, so I went up with the rail and I just sort of peeped my head around the bedroom doors without getting off the stairs. I daren’t stand up on the top step and walk. When I told them, they went mad they said I hadn't got to go up again. They thought I would fall down. So, I had strict instructions I hadn't got to up the stairs.’* (Participant 14, female, moderately frail, age 80s). ** Burden** •*‘You don't tend to pass the problem onto somebody else simply because you don't want to burden other people…because it means then that now they have got a problem they didn't need, they didn't want.’* (Participant 25, male, moderately frail, age 70s).
**Interpersonal level—unmet needs**
** Listening, empathy and rapport** • *“We have enough to go through without people treating us like we are nothing… You have got to be able to put yourself in somebody else’s shoes. I am like I have got loads of problems and you don't, so you don't know.”* (Participant 3, female, vulnerable, age 40s). •* ‘We have that kind of relationship between me and my GP. He’s very good at listening and deciding. Sometimes he’ll suggest things that I wouldn’t have thought of.’* (Participant 16, male, vulnerable, age 50s). • *‘The nurses, the medical staff here are very good…we see them three times a week, so we know them on the first names we know who has got a child and what the child’s name is. Do you see what I mean, we know their home life. Rapport is important…. it came down from [the top] that we should be made to feel valued.’* (Participant 10, female, vulnerable, age 50s). ** Unresponsive care** • *‘The doctor decides what will happen if there is a problem and every three months I have a clinic and they get the report on the computer and they decide what tablet I am going to take.’* (Participant 18, female, vulnerable, age 60s). • *‘When I told them I had a fall they didn't want to know. They said…you are perfect, your levels are perfect.’* (Participant 6, male, vulnerable, age 50s). ** Deferential relationships** • *‘I just go by what they say when they tell me.’* (Participant 23, female, moderately frail, age 60s). • *‘If they [the doctor] felt it probably was helpful for me then they would have done it a long time ago.’* (Participant 11, male, severely frail, age 60s).

### Factors contributing to frailty

Participants described living with a range of multiple long-term conditions, particularly diabetes and cardiovascular disease, in addition to their kidney condition. This cardio-renal-metabolic disease cluster gave rise to interlinking symptoms of weakness, exhaustion, pain and sleep disturbance. Weakness was felt throughout the body and contributed to a constant, yet variable, state of exhaustion. Participants described sleeping more frequently, but rarely experiencing refreshing sleep. Lack of sleep was also perpetuated by pain, arising from chronic musculoskeletal impairments, diabetic neuropathy, falls and HD procedures.

Multiple long-term conditions and their symptoms required complex medication regimes. Some participants self-medicated, including one who used recreational drugs. The majority stressed that there were few effective pharmacological options for managing pain. A few had independently explored non-pharmacological pain management strategies.

Most participants had also experienced weight loss but could not identify when this had occurred because of fluctuations in interdialytic fluid. They attributed weight loss to varying appetite (due to symptoms, illness, exhaustion and altered taste), difficulties physically preparing and eating food, and changed eating habits arising from emotions and forgetfulness. Taken together, participants’ accounts illustrate the multiple interrelating factors that contribute to their experience of frailty.

### Living with the consequences of frailty and HD

#### Individual level

Frailty, in addition to HD treatment, meant that most participants described a variable and unpredictable ability to undertake daily tasks, and to concentrate, rendering it necessary to live ‘day to day’. In addition to poor standing tolerance, walking, navigating steps and stairs, and transfers (such as sit to stand) were difficult. For some, recurrent vascular access infection, blockage, and aneurysm had resulted in a loss of upper limb function. A smaller number actively limited the use of their arm to preserve their vascular access. Combined, these led to difficulties with washing, dressing, and cooking. Initiation of HD and repeated hospital admissions were blamed for instigating and perpetuating reductions in ability and function. The culmination of these physical and practical impacts created a sense that life as planned had been interrupted. Participants described having to adjust to numerous losses, especially loss of confidence, motivation, independence, and social connection. Associated with these losses was a sense of grief, frustration, and sadness.

#### Interpersonal level

Almost all participants needed a diverse range of support from family members. This was predominantly practical (e.g., transport, preparing meals, shopping, personal care, accompanying to social events) but also included emotional and self-management support (e.g., acting as an advocate). The dynamics of friendship circles were altered by illness and loss of previous roles. Contact with friends diminished over time leading to loneliness and isolation, particularly for those without family.

#### Organisational level

Participants’ accounts revealed frustrations with trying to navigate a healthcare system not designed for people on HD, living with frailty and multi-morbidity. Participants and their families spent a lot of time arranging, chasing, and keeping track of multiple appointments from different clinics for different conditions, which was further complicated by thrice weekly attendance at HD. If appointments clashed, they had to be re-arranged, creating delays accessing care. Organising transport was also difficult and hospital transport was perceived to be unreliable.

Across all services, participants felt there was insufficient time to discuss their interacting conditions and psychological health or to address reablement sufficiently and consistently. Even within each service, participants described a lack of healthcare professional (HCP) continuity, resulting in duplication, inconsistent and conflicting care, and less opportunity to establish a rapport. Poor communication between services created delays when the relative risks and benefits of a treatment needed to be discussed between several specialities. In these circumstances, participants described living for extended periods with pain and uncertainty. Often, they reported confusion over which service was responsible for addressing which health issue and expressed a preference for kidney services dealing with their combined health problems. Some participants voiced concerns that these challenges were exacerbated by healthcare budget cuts.

#### Community level

Participants’ connection with their communities had shrunk over time, not only due to the restrictions imposed by the dietary, fluid and treatment requirements of HD and their experiences and consequences of frailty, but also accessibility-related barriers, and the perceived views of others in the wider community. Participants described feeling stigma from no longer conforming to the norms of their community. This loss of community participation created an enduring sense of loneliness. Some participants (all vulnerable to frailty) described how roles within community faith groups and voluntary organisations had provided a sense of purpose and wellbeing and an opportunity for being active and engaging with others. For the frailest, attending HD was the only time that they interacted with others, and was therefore an important source of social contact, despite the paradox that this contributed to their wider isolation.

#### Societal level

Participants’ dependency on others typically meant increased reliance on state benefits^1^ and support from voluntary organisations, but highlighted the challenges in acquiring these, not least due to changes and reductions to these in recent government policy. Accessing such support and other aid (e.g., disabled parking badges) was reportedly overwhelming. Assessments for benefits were described as stressful and inflexible, focusing solely on whether someone could complete a task and ignoring any subsequent symptomatic impact. Participants reported difficulty finding skilled support to navigate the benefits system, some felt forced to prioritise other living expenses above paying for support. A few attributed these challenges to efforts to reduce national expenditure on care and benefits.

### Coping strategies and unmet needs

#### Individual level

Participants used a range of strategies to cope. Problem-focused strategies were frequently mentioned, particularly avoidance of difficult activities and situations and vigilance, characterised by overplanning and monitoring for danger. Participants also mention protective mechanisms including “slowing down,” reducing their activity and replacing active hobbies with more sedentary ones. Adaptation was also common and took the form of creative problem solving, practically modifying one’s house, or accepting walking aids. Emotion-focused coping primarily manifested as resignation—a passive acquiescence to the undesirable, but assumed inevitable, consequences of frailty- or acceptance – the acknowledgement of the difficulties faced and adjustment of one’s mind-set. Both the use and success of these coping strategies fluctuated; a strong desire to retain or regain their independence meant participants often swung between avoidance and persevering with difficult tasks, impacting on symptoms and emotions.

#### Interpersonal level

Responses from both family and HCPs that were intended to be supportive, were often experienced as less helpful; some family members were described as risk-averse and ‘protective’ which manifested as rarely leaving the participants alone, being over-vigilant or encouraging avoidance. Participants felt some staff were afraid of them falling in hospital or at the HD unit, so discouraged activity. All participants were concerned about the physical and psychological burden that a caring responsibility created for those helping them. Some had the additional worry that family members were living with their own health concerns or caring for several people within the extended family and had support needs of their own. Relationships were sometimes strained or co-dependent. These factors led participants to withhold information and project a positive disposition in an effort not to “bother” their family. Together, perceived family overprotection and participants’ desire not to be a burden reinforced avoidant behaviours and negatively impacted upon participants’ independence, ability to participate in community activities and their role within the family.

Helpful HCP support included listening and empathy, which were seen as key to developing a relationship and a rapport. Such relationships made participants feel valued, engendered confidence, and built trust, in turn helping them to broach sensitive issues (e.g., psychological health) and to feel safe while receiving care. However, around half of the participants described the care they received as unresponsive to their needs, addressing acute problems only, e.g., reacting to blood results or completing specific care processes. They sometimes felt that their concerns were brushed aside, and symptoms and falls were not taken seriously. Those who appeared to be managing their condition reported not being asked about support needs.

Most participants indicated being deferential to HCPs and sometimes reluctant to speak up, ask questions and seek clarification or information. Some believed that if a treatment or course of action were warranted, the HCP would suggest it, while others feared appearing ‘silly’ or unwanted consequences if they did speak up. For example, while most participants were keen on regular medication reviews, those with chronic pain were wary about the consequences of any reduction. A few participants described working in partnership with the HCP, taking an active role in learning about their condition, and suggesting courses of action, although this did not necessarily result in effective self-management strategies being implemented.

## Discussion

This study aimed to explore the lived experiences of people receiving HD who are frail and to identify which factors should inform the holistic care of this group. To summarise, the main factors which participants described as contributing to frailty were weight loss, weakness, exhaustion, pain, and sleep disturbance arising from multiple long-term conditions. These issues magnified the losses and challenges associated with HD treatment by necessitating more frequent and burdensome interactions with health and social care organisations, which did not always prioritise their needs. Participants were increasingly reliant upon family members and vulnerable to loneliness due to diminishing social support and community participation. In response, some participants were able to adapt to their circumstances by adopting creative problem solving and embracing aids and adaptations which promoted independence but for others avoidance, vigilance and resignation were their primary coping strategies. Reinforced by family and healthcare professional relationships, these led to escalating dependence and a reduced ability to engage in self-management behaviours and decision-making. Overall, the findings suggest that interventions and care pathways that work to address the factors identified across multiple socioecological levels may result in broader improvements in both experience and outcomes for this group.

The experiences of this patient group who are receiving HD *and* living with frailty echo – to an extent – findings from studies of older people living with end-stage kidney disease and those living with frailty in the general population [16, 17, 21, 29, 30]. These studies highlight the impact of symptoms [17, 30], multiple long-term conditions [21], a resulting loss of social life [17, 30] and increasing reliance on family members [17, 21, 29, 30] and coping primarily via acceptance [17, 29, 30], problem solving but also psychological avoidance [17]. Our findings extend these by illustrating that HD and frailty are *both* disruptive life events that, when combined, have a cumulative impact which profoundly unsettles people’s expected life course, irrespective of age. By drawing upon the socioecological model to present our findings, we demonstrate that the consequences of living with HD and frailty were evident at multiple levels and we highlight the corresponding unmet needs of this patient group. The multi-level consequences and needs underline the potential importance of embedding a more holistic assessment in kidney services which could identify unmet needs at each level; for example, at the individual level, assessing symptom and medication management, nutrition, function and mobility and psychological wellbeing; and at the interpersonal level, assessing amount and type of social support. Our results suggest that such an assessment may be particularly useful upon admission to hospital and, for those who are considering dialysis options, in the outpatient setting, to inform decision-making and prompt the introduction of treatment strategies which may prevent or allay decline.

One form of holistic assessment relevant to this population is Comprehensive Geriatric Assessment (CGA). CGA is a multidimensional, multidisciplinary process which identifies holistic care needs and leads to the development of an integrated and co-ordinated care plan [13]. In older people within the general population, when compared to acute general medical care, those who receive CGA are more likely to be alive and in their own homes on discharge and at 3 to 12 months' follow‐up [31]. CGA is not yet a routine part of kidney services. Barriers to implementation are not well established within the literature, but include uncertainty about how to identify and target suitable recipients [18, 32], lack of time and knowledge [18, 12], concern about participant burden [18] and inconsistent access to a broad multi-disciplinary team [12]. Whilst it is not yet known if CGA leads to improved outcomes in people with chronic kidney disease, existing evidence suggests that CGA can successfully be integrated, in a modified format, into routine kidney care, leading to improved experience, identification of geria tric impairments, and initiation of discussion around treatment decisions [11, 12, 33–35]. These modified versions of CGA are typically completed by one or two assessors (usually a nurse and/ or nephrologist) who conduct a streamlined version of the CGA. The content of the assessment varies, but most commonly focuses upon mobility and function, falls, nutrition, cognition and the presence of anxiety and depression [11, 12, 33–35]. Our results indicate that widening this assessment to identify additional needs (relating to symptom management, physical inactivity, self-management support, benefit assistance and coping with loss, grief, and loneliness) would be of particular help to the frail HD population. In addition, involving other speciality services in these holistic assessments, and potentially utilising telehealth and virtual multi-disciplinary team meetings could potentially overcome issues of duplication and inconsistency in care that participants described.

At the interpersonal level, family were providing extensive support, invariably leading to conflict between a family’s desire to ‘protect’ and a participant’s desire to avoid being a ‘burden’, which increased avoidant behaviours and limited their independence. These findings echo previous research that highlights the influence of family support on individual’s confidence and ability to manage their health [16–18, 21]. To address this, kidney services could develop better mechanisms to support family and friends caring for those with frailty, including the development of family-based supportive interventions, and greater involvement of family members in decision-making, acknowledging of course, that the person must remain at the centre of their care [9, 36–38].

The findings of this study underline that assessment and intervention at the individual and interpersonal levels may be insufficient in producing sustained improvements in outcomes and care for this group. At an organisational level, lack of time, continuity and accommodations for multi-morbidity and complexity within services led to an experience of transactional care, which did not always meet patients’ needs. Furthermore, deferential attitudes towards healthcare professionals led to participants under-reporting issues important to them, such as symptoms and falls, and the undermining of the therapeutic alliance needed for effective self-management. Previous research has highlighted similar experiences in the general HD population [16, 18]. Our findings highlight the importance of person-centred care, which focuses upon the individuals experience of their illness, but we note the difficulties of achieving this in the context of a pressurised health and social care system [38]. A focus upon guideline-driven care, which prioritises clinical targets, has been linked with discordant treatment recommendations and communication between care providers and patients [17, 37, 38]. Efforts to streamline and enhance the co-ordination of care across sectors has been linked to increases in function, patient, carer and HCP satisfaction and reduced institutionalisation, our findings call for work to enhance the integration between kidney services and other health and social care services [39].

This study emphasises the dynamic interactions between the factors contributing to frailty and its consequences, strategies for managing frailty and unmet needs, at multiple socioecological levels. The development of close collaborative links between kidney services and other secondary and primary care specialities, community groups and integrated care systems may also, therefore, facilitate developing and evaluating multi-level interventions, which could lead to better outcomes and experiences than interventions solely targeting the individual level [40, 41]. For example, a more sustained impact may be achieved by combining: a holistic assessment and management plan; family support interventions; implementation of person-centred care pathways; increased links with groups and services; enhanced accessibility of/to community environments; and reform to state financial support systems. In addition, while it is difficult for interventions to address rapport directly, particularly given the pressures on staff, a more integrated approach to care could increase consistency and time and, in turn, this may enhance trust, empathy and rapport.

To our knowledge, this study is the first to explore the experiences of people living with frailty *and* receiving HD. A key strength was the use of a validated frailty risk-stratification measure to identify those living with frailty. Despite this, the study inclusion and exclusion criteria potentially limited the transferability of our findings. Recruiting participants living with severe frailty (CFS 7) was challenging, although the proportions included are comparable to the HD populations [42]. A further limitation of this study was the exclusion of non-English speakers, however the inclusion of an ethnically diverse range of participants is a particular strength of this work. Patient and public involvement during the design, data collection, analysis, and interpretation phases of the study also helped to ensure both rigour and credibility.

The lead author is a physiotherapist working with people with long-term conditions, and this may have shaped the data collection process and the themes constructed. Some of the participants were also known to her in her therapy role, which may have influenced their responses. She maintained a reflexive journal throughout the study, to document reflections on her potential influence on the research process and the interpretation of the findings, and to create an ‘audit trail’ of analytical decisions. The involvement of N.R.(a senior renal dietitian), M.H. (a diabetes specialist nurse), and H.E. (a social scientist) in the analysis and interpretation of the data enabled us to reflect upon the level of agreement and similarity between coders and to ensure that analysis and interpretation was grounded in the data. To enhance credibility, we discussed our findings with the wider multidisciplinary research team and our PPI group.

## Conclusion

In conclusion, frailty and multiple long-term conditions magnify the losses and challenges associated with HD treatment, demanding more frequent and burdensome interactions with health and social care services for the individual and their families. The implementation of CGA into kidney services, designing health and social care systems which facilitate the development of more person-centred approaches and relationships, greater inclusion of family members and enhancing opportunities for community participation may all improve outcomes and experience. However, an approach which addresses all these areas, in addition to wider public health interventions, may have greater sustained impact. 

## Supplementary Information

**Additional file 1: Supplementary material 1.** Topic guide outline for interviews.

## Data Availability

The datasets used and/or analysed during the current study are available from the corresponding author on reasonable request.

## Abbreviations

CFS: Clinical Frailty Scale
CGA: Comprehensive Geriatric Assessment
COREQ: Consolidated criteria for reporting qualitative research
HCP: Healthcare professional
HD: Haemodialysis
PPI: Patient and public involvement
IQR: Interquartile range

## Footnotes

1 In the UK people who need financial support can receive this in the form of benefits and tax credits provided they meet certain criteria. For more information see https://www.kidneycareuk.org/about-kidney-health/living-kidney-disease/benefits/
