# Supplementary material for: Living with frailty and haemodialysis: a qualitative study
Source: BMC Nephrol. 2022 Jul 22;23:260. doi: 10.1186/s12882-022-02857-w (PMC9308309; doi:10.1186/s12882-022-02857-w)
Supplement: Supplementary file 1 — Additional file 1: Supplementary material 1. Topic guide outline for interviews. [file 12882_2022_2857_MOESM1_ESM.pdf]

Supplementary material 1: Topic guide outline for interviews

1. To begin, can you tell me a little bit about how you came to be on dialysis?

2. Has dialysis affected you physically?

Prompts:

- -Weakness,
- -Slowness,
- -Exhaustion,
- -Levels of physical activity
- -Unexplained weight loss

3. Has dialysis affected you in any other ways?

Prompts – changes/challenges to:

- Daily routine
- Health, other long term conditions
- Interactions with healthcare professionals
- Needing help with ADLS/finance/medication
- Social interactions and hobbies
- Cognition, concentration and memory
- Psychological – happiness, loneliness, emotion, outlook on life, confidence

4. One of the reasons I asked you to take part in this study is because you have had a fall in the past. Can you tell me about what happened when you last fell?

Prompts:

- Do they remember?

- dizziness/ unsteadiness/ trips and slips/ vertigo/ syncope
- Activity at time?
- Direction of fall?
- Where? Probe into environment factors involved
- Time of day?
- Alone or accompanied?
- Injury?
- Able to get up?
- Pendant alarm

5. What do you think caused you to fall over?

- Environment (home, dialysis, outside)
- Post dialysis effects
- Cardiac impairment
- Balance impairment
- Neurological cause
- ENT impairment
- External force
- Lack of activity/ weakness
- Pre-existing health problem
- Medication

Did others have different ideas of cause?

6. What happened immediately after you fell?

- What self-care did they undertake?
- Healthcare utilisation/ contact with health professional or other service

- Family response/ impact
- Social impact / impact on daily life and routine
- Emotional impact / impact on confidence
- Physical impact – health/ activity levels/ function

7. What happened because of the fall in the longer term?

- Same prompts as question 6

8. Did you discuss this fall anyone at the time?

Prompts:

- If yes – who[Healthcare professionals, family friends etc.]? Who initiated this and why, what discussed, with whom, outcome?
- If no – why not?

9. Were you offered any support or treatment after you fell?

- Yes – what support was offered?
- Thoughts on what was offered/ advised/ suggested?
- Did they take it? Outcome? Any challenges to participating?
- No/ not take – why not? What do you think would have been useful?
